# Idiopathic pulmonary fibrosis and coronary artery disease

**DOI:** 10.1186/2049-6958-9-31

**Published:** 2014-05-30

**Authors:** Gaetano Cicchitto, Valentina Musella, Maria Acitorio, Nicola Capuano, Giuseppe Fiorenzano, Caroline A Owen, Mario Polverino, Francesca Polverino

**Affiliations:** 1 Pulmonary Division Unit, Cava de’ Tirreni, (SA), Italy; 2 Cardiology Unit, Nocera, (SA), Italy; 3 Internal Medicine Department, Pulmonary Division Unit, Terni, Italy; 4 Division of Pulmonary and Critical Care Medicine, Brigham and Women's Hospital, Harvard Medical School, Boston, MA, USA; 5 Lovelace Respiratory Research Institute, Albuquerque, NM, USA

**Keywords:** Cardiopulmonary exercise test, Coronary artery disease, Idiopathic pulmonary fibrosis

## Abstract

Idiopathic pulmonary fibrosis (IPF) is defined as a chronic fibrosing interstitial disease of unknown cause, limited to the lungs, and associated with the histopathologic and/or radiologic pattern of usual interstitial pneumonia (UIP); it generally progresses into respiratory failure and death. Although progression of the disease is the most common cause of death, there are increasing reports of its association with other pathologies has been reported: e.g., IPF patients seem more susceptible to cardiovascular diseases. Therefore, other pathologies might also influence the natural course.

In this paper, we describe a case of IPF and coronary artery disease (CAD). We emphasize the importance of cardiopulmonary exercise test (CPET) as a useful procedure to monitor disease progression in IPF patients. We also stress the importance of a careful analysis of variables measured for an accurate interpretation of the clinical picture and an improvement of the clinical management of patients. Moreover, we suggest that a careful assessment of CPET parameters may additionally help in the early detection of high cardiovascular ischemic risk.

## Background

Idiopathic pulmonary fibrosis (IPF) is defined as a form of chronic, progressive fibrosing interstitial pneumonia of unknown cause, occurring primarily in older adults, limited to the lungs, and associated with histopathologic and/or radiologic pattern of usual interstitial pneumonia (UIP) [1]. People with IPF usually die because of progression of their underlying lung disease [2], even if there is evidence of increased incidence of other diseases, such as lung cancer [3,4], which could affect the time of death. Furthermore, IPF patients seem more susceptible to developing coronary artery disease (CAD) than people with non fibrotic lung disease or other non-IPF pulmonary fibrosis [5]. Cardiopulmonary exercise test (CPET) is widely employed in patients with heart/lung diseases to study the level of exercise tolerance and to define either preoperative risk for thoracic surgery, or the response to pharmacological therapy and to rehabilitation programmes. For this purpose a cluster of parameters are used [6,7]: work efficiency (WR, work rate), metabolic response (VO_2_, oxygen uptake; VCO_2_, carbon dioxide output; AT, anaerobic threshold), ventilatory/respiratory response (TV, tidal volume; Rf, respiratory frequency; VE, minute ventilation;VE/VCO_2_ and VE/VO_2_ ratios, ventilatory equivalents; VD/VT, ratio of physiologic dead space to tidal volume), and cardiovascular response (HR, heart rate; VO_2_/HR, oxygen-pulse; ΔVO_2_/ΔWR, change in the oxygen uptake related to the increase in work rate), PaO_2_, PaCO_2_, pulmonary gas exchange (arterial oxygen and carbon dioxide partial pressures; SaHbO_2_, arterial O_2_ saturation). CPET is rarely of diagnostic use in patients with respiratory or cardiovascular dissease. However, it can aid in the clinical management of patients with established cardiopulmonary diseases.

In this paper we describe a case of IPF and CAD, emphasizing the role of CPET in early diagnosis of CAD.

## Case presentation

**Figure 1:**
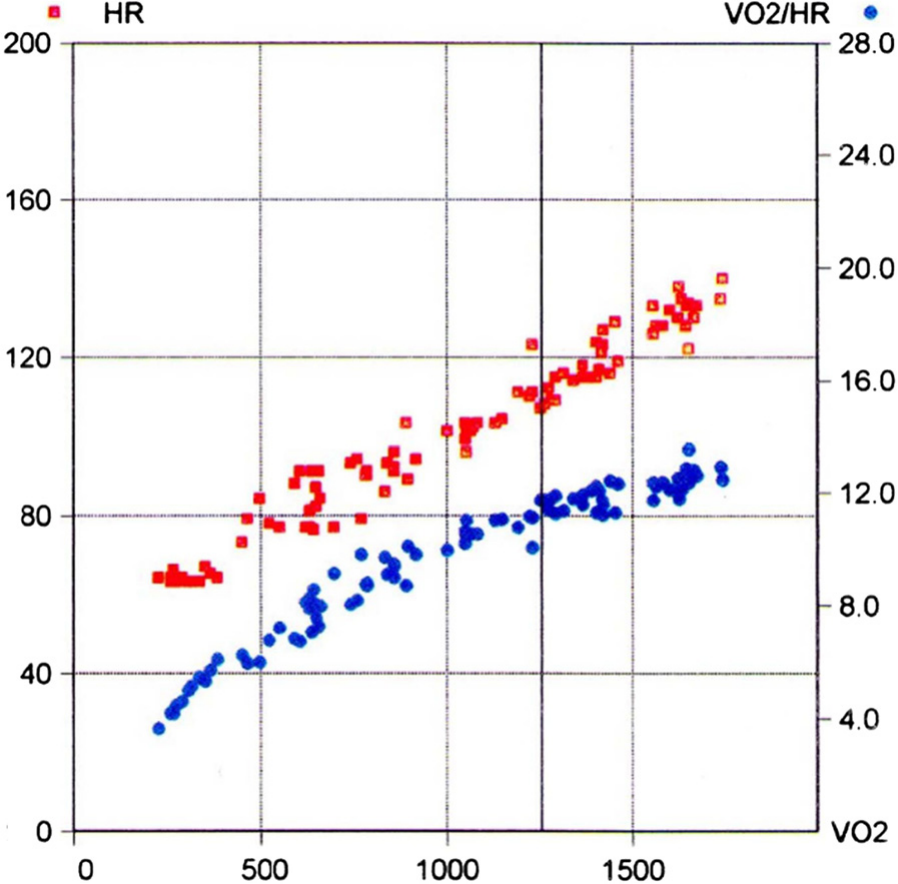
**Oxygen pulse (VO**_**2**_**/HR) at the time of the diagnosis.** x-axis: VO_2_; left y-axis: heart rate (HR) represented by red dots; right y-axis: VO_2_/HR represented by blue dots.

A 60-yr old Caucasian man was admitted to the hospital due to a dry cough and progressive dyspnoea on exertion. The patient reported a history of smoking, but no systemic disease, or occupational exposure, and was not taking any medications. Physical examination revealed bilateral basal inspiratory crackles. A High Resolution Computed Tomography (HRCT) scan showed basal distribution of reticular opacities, traction bronchiectasis and possible honeycombing. Pulmonary function tests (PFT) were abnormal as spirometry indicated restriction (TLC 76%, FVC 97%, FEV_1_ 102%, FEV_1_/VC 76.9%), reduced diffusing capacity for carbon monoxide (DL_CO_ 77.5%), with a preserved ratio of DL_CO_/VA (111%). Bronchoalveolar lavage (BAL) sampling revealed a mild granulocytic alveolitis (88% macrophages, 5% lymphocytes, 5% neutrophils and 2% eosinophils) and reduced lymphocytic subpopulation ratios (CD4/CD8 0.5). Arterial blood gases were normal under resting conditions (pH 7.38; PaO_2_ 90 mmHg; PaCO_2_ 42 mmHg; SaO_2_ 97%), but not during exercise, when mild hypoxemia was seen (PaO_2_ 60 mmHg). Neither an echocardiographic examination nor a CPET showed pathological signs. Instead, normal work efficiency, metabolic, ventilatory, and cardiovascular responses were obtained; in particular, a linear increase in VO_2_, together with an increase in work rate above the anaerobic threshold, was found. Due to diagnostic uncertainty, three months after the previous assessment the patient underwent a complete functional reassessment including a renewed CPET, which did not show any relevant changes. The patient was then subjected to a thoracoscopic lung biopsy (Video-Assisted Thoracoscopic Surgery, VATS) which revealed a pattern of UIP, thus confirming the IPF diagnosis. In order to monitor the course of the illness, the patient was closely monitored with additional clinical and physiologic testing. In this context, two months after performing a surgical lung biopsy, the patient repeated PFTs and an echocardiographic study. However, these investigations did not show significant changes from the previous two evaluations, except for a mild reduction in resting oxygenation (PaO_2_ 70 mm Hg). However, analysis of CPET, in spite of normal metabolic and ventilatory responses, showed an abnormal cardiovascular finding relative to the pattern of increased increase of increase VO_2_ relative to work rate below and above anaerobic threshold (Figure 1A): the ΔVO_2_/ΔWR ratio was reduced from 11.3 ml/min/W to 6.9 ml/min/; furthermore, changes both in oxygen-pulse (Figure 1B) and in electrocardiographic signs that could be due to different causes were observed. The patient was referred to a Cardiology Unit, where a diagnosis of acute coronary syndrome (ACS) was made and percutaneous transluminal coronary angioplasty (PTCA) was performed.

**Figure 2:**
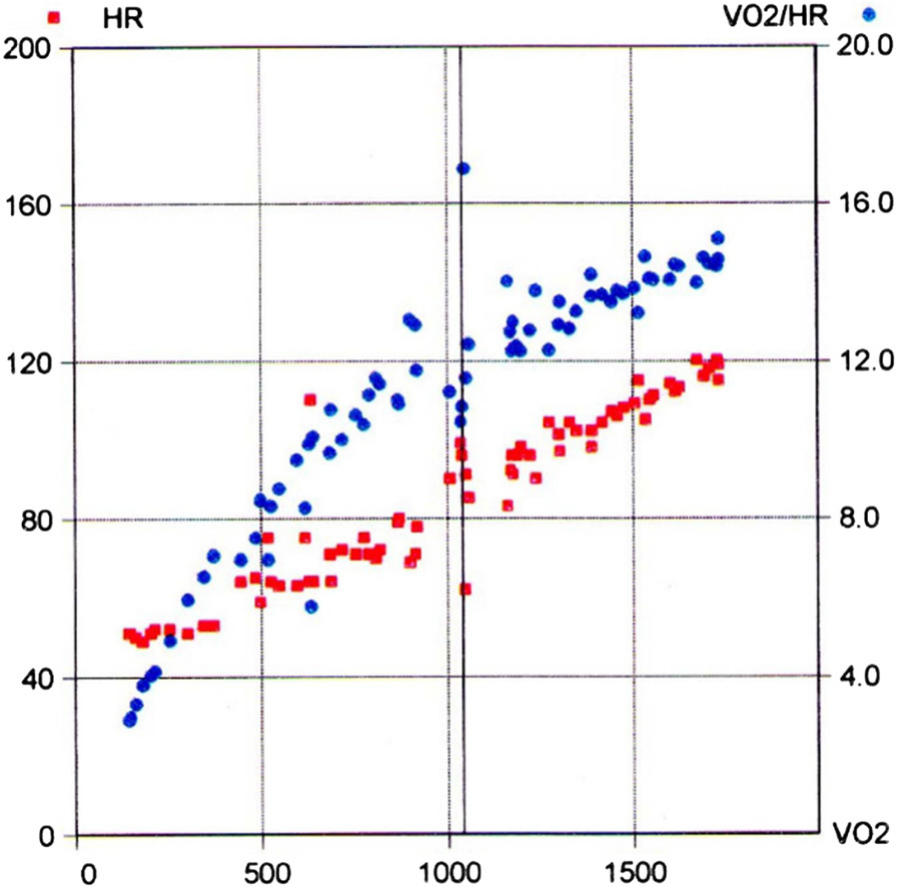
**Oxygen pulse (VO**_**2**_**/HR) after cardiac therapy.** x-axis: VO_2_; left y-axis: heart rate (HR) represented by red dots; right y-axis: VO_2_/HR represented by blue dots.

Six months after the patient’s referral to the Cardiology Unit, another CPET showed a totally normal result both with respect to the ΔVO_2_/ΔWR ratio and the oxygen-pulse (Figure 2).

## Discussion

CPET is a well accepted technique utilized in clinical practice to evaluate cardiovascular and respiratory interactions during physical exercise. It is not routinely used in the management of IPF patients but, when performed, some parameters may have prognostic significance [8,9]. In addition, although IPF, by definition, is a progressive chronic fibrosing disease limited to the lung, the heart might be involved when gas exchange is so altered that coronary flow is affected. In this context, CPET could be a useful tool to investigate subclinical CAD [10]. While the progression of the underlying lung disease is the most common cause of death for patients with IPF [2], various studies seem to suggest an additional increase in cardiovascular diseases in this population [11], which could have an impact on morbidity and mortality. In spite of this, IPF guidelines do not recommend routine screening to evaluate the presence of cardiovascular complications, as it is not clear whether treating comorbidities may influence survival [1]. However, in the context of comprehensive care of IPF patients, an holistic approach was recently proposed [12], in which identifying and treating comorbidities, such as CAD, could be a strategy to optimize the management of the disease. In the cardiological context, CPET is a well recognized method to diagnose and/or investigate cardiovascular dysfunction (e.g., myocardial ischemia, chronic heart failure) [10,13]. With respect to lung diseases, the goal of CPET is the identification of physiopathological determinants of respiratory dysfunction as well as matching the pattern of the abnormality with the particular sites of the system involved, thus clarifying the clinical picture and possibly narrowing the differential diagnosis [14,15]. Furthermore, the physiological link between respiratory and cardiovascular functions might cause the changes in one system to influence the performance of the other. That is, deterioration in gas exchange could be the cause of CAD, which could manifest as changes in the slope of ΔVO_2_/ΔWR and a flattening of the oxygen-pulse [16]. In previous studies, various indices of exercise capacity have been evaluated as prognostic factors for IPF [9,17], in particular VO_2_-max [18]. Moreover, they were shown to be significantly related to prognosis when baseline values and percent variation over time were assessed. Although the results of these investigations were not concordant [19], due at least partially to the employment of different methodologies, gas exchange impairment during exercise has generally been emphasized as a factor influencing clinical progression of disease in IPF patients.

In our clinical practice, we perform PFTs on IPF patients to monitor progression of the pulmonary disease and to obtain data facilitating its management, such as the optimal time for referral of the patients to a lung transplantation center. Whenever possible, we use CPET to detect early changes in pulmonary physiology, especially gas exchange, and to verify the presence of cardiovascular abnormalities.

We hereby confirm the importance of CPET as a useful procedure to monitor disease progression in IPF patients. CPET can be useful in differentiating whether the worsening of dyspnoea leading to exercise limitation is related to pulmonary or cardiovascular causes. Discriminating between these two possible causes of dyspnoea worsening can be useful in the decision-making process during the management of the patient (e.g., for inclusion in clinical trials and transplantation referral). Furthermore, we stress the necessity of a careful analysis of variables measured for an accurate interpretation of the clinical picture. In particular, we consider that an in-depth study should be started even when slight and/or uncertain responses are obtained from the analysis of involved variables, and even if uncertainties occur in only one of several relevant parameters. In the case described herein, we requested a cardiac workup soon after the time of the initial diagnostic suspicion of CAD. This resulted in an earlier clinical diagnosis and earlier initiation of treatment of the patient.

## Conclusions

The conclusion that might be drawn from this report is that a more extensive CPET should be performed for better management of IPF patients, not only with the goal of identifying useful prognostic parameters, but also to detect potentially treatable cardiovascular alterations earlier.

The careful assessment of CPET parameters (e.g., O_2_ pulse and work efficiency above and below the anaerobic threshold) may not only improve clinical management of IPF patients, but also identify factors predisposing to higher ischemic risk. However, further studies are needed to assess the costs and benefits of associated with generalized use of CPET in the care of IPF patients.

## Consent

Written informed consent was obtained from the patient for the publication of this report and any accompanying results.

## Competing interests

The authors declare that they have no competing interests.
